# Alcohol abuse increases the risk of HIV infection and diminishes health status of clients attending HIV testing services in Vietnam

**DOI:** 10.1186/s12954-016-0096-z

**Published:** 2016-02-16

**Authors:** Bach Xuan Tran, Long Hoang Nguyen, Cuong Tat Nguyen, Huong Thu Thi Phan, Carl A. Latkin

**Affiliations:** Institute for Preventive Medicine and Public Health, Hanoi Medical University, Hanoi, Vietnam; Johns Hopkins Bloomberg School of Public Health, Baltimore, MD USA; School of Medicine and Pharmacy, Vietnam National University, Hanoi, Vietnam; Institute for Global Health Innovations, Duy Tan University, Da Nang, Vietnam; Authority of HIV/AIDS Control, Ministry of Health, Hanoi, Vietnam

**Keywords:** Alcohol, Sex, Drug use, AUDIT-C, HIV testing, Vietnam

## Abstract

**Background:**

Vietnam is among those countries with the highest drinking prevalence. In this study, we examined the prevalence of alcohol use disorders (AUDs) and its associations with HIV risky behaviors, health care utilization, and health-related quality of life (HRQOL) among clients using voluntary HIV testing and counseling services (VCT).

**Methods:**

A cross-sectional survey of 365 VCT clients (71 % male; mean age 34) was conducted in Hanoi and Nam Dinh province. AUD and HRQOL were measured using the Alcohol Use Disorders Identification Test-Consumption (AUDIT-C), and EuroQol-five dimensions-five levels (EQ-5D-5L). Risky sexual behaviors, concurrent opioid use, and inpatient and outpatient service use were self-reported.

**Results:**

67.2 % clients were lifetime ever drinkers of those 62.9 % were hazardous drinkers and 82.0 % were binge drinkers. There were 48.8 % respondents who had ≥2 sex partners over the past year and 55.4, 38.3, and 46.1 % did not use condom in the last sex with primary/casual/commercial sex partners, respectively. Multivariate models show that AUD was significantly associated with risky sexual behaviors, using inpatient care and lower HRQOL among VCT clients.

**Conclusions:**

AUD was prevalent, was associated with increased risks of HIV infection, and diminished health status among VCT clients. It may be efficient to screen for AUD and refer at-risk clients to appropriate AUD counseling and treatment along with HIV-related services.

## Background

Vietnam is among those countries with the highest drinking prevalence in the world [1]. Drinking alcohol is legally and culturally accepted in Vietnamese traditions, especially among men [2, 3]. A nationally representative survey revealed that 80 % of males drank alcohol and 40 % were hazardous drinkers compared to under 5 % of female [4]. In the rural of Vietnam, 87.3 % male and 10.2 % female reported drinking alcohol in the last 12 months [5].

Among people living with HIV (PLWH), high alcohol consumption is associated with treatment non-adherence and poor immunological and viral outcomes [6, 7]. In addition, drinking alcohol is associated with physical and mental problems such as neuropathy pain [8], lipodystrophy [9], and depression [10]. In Vietnam, alcohol consumption and alcohol use disorders (AUDs) have been found to negatively affect adherence to HIV medication and health-related quality of life (HRQOL) of patients with HIV/AIDS [2, 3, 11, 12]. However, the relationship between alcohol use and higher HIV risk behaviors has not been studied. In many other settings, alcohol abuse and its relations to HIV transmission have been well documented [13–18]. Heavy alcohol drinkers were more likely to engage in unsafe sexual behaviors, have multiple sex partners, or share syringes that increased the risk of HIV and other sexual transmitted infection (STI) transmissions [19–23].

The HIV epidemic in Vietnam is still in a concentrated stage, which is mostly driven by at-risk populations such as people who inject drug (PWID) and female sex workers [24] [25]. Although unsafe drug injection has been a predominant mode of transmission, there has been a significant increase of new cases got infected with HIV by unsafe sex [24]. Several studies have revealed socioeconomic and geographical differences that shape health behaviors as well as access, use, and outcomes of HIV/AIDS interventions in Vietnam. Kaljee et al. (2005) found that alcohol use is associated with intention and engagement in sexual behaviors among rural adolescents [26]. Nguyen et al. (2014) found higher odds of drug use among those motorbike taxi drivers who were born in urban cities, currently residing in rural areas, and using alcohol [27]. In addition, patients in the rural and urban perceived quality of care and reported health-related quality of life outcomes disproportionally [2, 28–32]. In the rapid urbanization of Vietnam, alcohol production and consumption has significantly changed. While urbanized people prefer branded products, mainly beer, the traditional alcohol drinking is common among older people in rural areas [33]. As a result, it is necessary to characterize how health behaviors and associated factors differ between urban and rural people to inform contextualized interventions in Vietnam.

Scaling-up HIV voluntary counseling and testing (VCT) service has been a priority of HIV/AIDS prevention programs in Vietnam [34]. In previous study, we founded that although only 4.1 % VCT clients were current opiate users, the prevalence of people having risky sexual behaviors was very high [35]. We hypothesized that AUD might be a significant predictor of unsafe sex and other health problems among MMT clients. In addition, these risk behaviors might vary across diverse socioeconomic groups, for example gender and location. The current study assessed alcohol use prevalence among VCT clients and examined associations between AUDs and sexual risk behaviors as well as HRQOL and health care service utilization among respondents.

## Methods

### Study setting and sampling

A cross-sectional study was conducted in Hanoi and Nam Dinh from January to August 2013. Six VCT clinics were purposely selected by the following criteria: (1) involve facilities at provincial, district, and commune levels [36, 37]; (2) implementing VCT services according to national guidelines [38]; and (3) consisting of both urban and rural areas. In Vietnam, commune health station is a “gate-keeper” that provides basic medical care and implements public health programs [36, 37]. To recruit participants, a recruiter invited clients from the selected VCT clinics to participate in the study.

Eligibility of sampling included (1) clients aged 18 or above, (2) initiating the service or returning for the test results, and (3) able and willing to answer questions and provided written informed consent. The interviews were conducted in a private room by either counselors at the VCT clinics or well-trained researcher staffs.

### Measures and instruments

A structured questionnaire was developed to examine alcohol use disorders and risk behaviors. We reviewed the literature to define factors that might be related to these outcomes of interest, including socioeconomic status, health status, history of drug use, and VCT service utilization [20, 22, 23]. In addition, we also assessed the interaction between alcohol use and gender [22].

#### Socioeconomic status

Information about age, gender, marital status, educational level, employment, religion, and household income were collected. Household income per capita was measured by estimating from all sources of income of all family members and then categorized into five quintiles: poorest, poor, middle, rich, and richest.

#### Health status

The EuroQol-five dimensions-five levels (EQ-5D-5L) instrument [39] was used to assess HRQOL of VCT clients. The Vietnamese version of this instrument has showed good measurement properties in this patient group [12, 32]. It includes five domains: mobility, self-care, usual activities, pain/discomfort, and anxiety/depression with five responses from “no problem” to “extreme problem.” By combining all domains and responses, a total health status index was created. Since the population’s norms for Vietnam are unavailable, we used the interim scoring of EQ-5D-5L from a cross-walk value set from Thailand [39]. In addition, a visual analog scale (EQ-VAS) was used to evaluate the self-rate health of participants on a 20-cm vertical scale. The score of EQ-VAS ranges (0; 100) from “the worst health you can imagine” to “the best health you can imagine.”

Additionally, we collected data about self-reported HIV status, which was categorized into three groups: HIV positive, HIV negative, and non-available or unknown (N/A).

#### Alcohol abuse

A brief instrument, the Alcohol Use Disorders Identification Test-Consumption (AUDIT-C), was used to screen heavy drinker and alcohol abuse [40]. The Vietnamese version of this tool was used and validated elsewhere [3, 41]. It consists of three questions contributing to a band score of (0; 12), with a higher score corresponding to a higher risk of alcohol dependence. If male respondents had score ≥4 and female respondents had score ≥3, they were categorized as hazardous drinkers [40]. The respondents were also classified as binge drinkers if the last item of the instrument received any positive response [42].

#### Sexual behaviors measure

Included information about whether clients ever had sexual intercourse, the number of sex partners in the last 12 months, types of sex partner (primary, casual, commercial), and condom use in the last sexual intercourse with each type of partner in the past 30 days.

#### Opiate drug use behaviors

Included information on historical and current drug use. Current drug use was defined if the clients used illicit opioid drug within the past 30 days.

#### VCT and health services utilization

We collected information about the frequency of VCT use, clients’ referrers, willingness to refer their partners or relatives to VCT, and willingness to be voluntary peer educators. Inpatient and outpatient health care utilization in the past 12 months were also collected.

### Statistical analysis

*P* value <0.05 was considered statistical significance. Student *t* test and *χ*^2^ tests were used to evaluate the difference of characteristics between resident areas (urban and rural). Multivariate logistic regression was employed to determine the associated factors with outcomes of interests, including “having multiple sexual partners in the last 30 days,” “non condom use in the last sex with primary/casual/commercial sex partners,” “outpatient services use,” and “inpatient services use.” Additionally, the range of EQ-5D index and EQ-VAS was left- and right-censored since their score ranged at (−0.452; 1) and (0; 100). The censored data limits the accurate estimation of values that are below and above the thresholds. Therefore, we used censored regression (Tobit) models to identify factors associated with EQ-5D index and EQ-VAS. The reduced model was constructed using stepwise backward selection, which was based on log-likelihood ratio tests with *p* value >0.2 as a threshold for exclusion.

### Ethical approval

The research was approved by the Scientific and Ethical Committee of the Authority for HIV/AIDS Control at the Vietnamese Ministry of Health.

## Results

Of 365 VCT clients, the mean age was 34.0 (SD = 8.4). There was no different between age and marital status between urban and rural respondents (*p* > 0.05). In urban areas, the proportion of male clients (71.1 %) was significantly higher than in rural areas (57.6 %) (*p* = 0.02). The employment status and education attainment were also significantly different between the two groups (*p* < 0.01).

Table 1 reveals that the proportions of people having any problem in mobility, self-care, and usual activities in rural were significantly higher than people in urban areas (*p* < 0.01), while the prevalence of having problem in anxiety/depression among urban clients (75.6 %) was much higher than those in rural areas (48.5 %) (*p* < 0.01). The mean EQ-5D index and EQ-VAS was 0.78 (SD = 0.16) and 85.6 (SD = 13.7), respectively. About 4.7 % were HIV positive, with no difference between urban (5.6 %) and rural (2.0 %) groups (*p* = 0.07).

**Table 1:** Alcohol use and health status of VCT clients in 2013 (*n* = 365)

	Rural		Urban		Total		*p*
	*N*	%	*N*	%	*N*	%	
Frequency of drinking alcohol							
Never	40	40.4	80	30.1	120	32.9	0.2
Never a month or less	24	25.3	65	24.4	90	24.7	
Never 2–4 times/month	20	20.2	69	25.9	89	24.4	
Never 2–3 times/week	13	13.1	40	15	53	14.4	
Never ≥4 times/week	1	1.0	12	4.5	13	3.6	
Amount of drinks containing alcohol on typical day							
Never ≤2	91	91.9	141	53.0	232	63.5	<0.01
Never 3 or 4	6	6.1	69	25.9	75	20.5	
Never 5 or 6	2	2.0	42	15.8	44	12.1	
Never 7 or 9	0	0.0	2	0.8	2	0.6	
Never ≥10	0	0.0	12	4.5	12	3.3	
Frequency of drinking ≥6 drinks on one occasion							
Never	67	67.7	97	36.4	164	44.8	<0.01
Less than a month	18	18.2	94	35.3	112	30.7	
Monthly	12	12.1	46	17.3	58	15.9	
Weekly	2	2.0	27	10.2	29	8	
Daily or almost daily	0	0.0	2	0.8	2	0.6	
Hazardous drinkers (# ever drinkers 245)	22	37.3	132	71.0	154	62.9	<0.01
Binge drinkers (# ever drinkers = 245)	32	54.2	168	90.9	201	82.0	<0.01
Health status							
Have problems in mobility	37	37.4	58	21.8	95	26.0	<0.01
Have problems in self-care	24	24.2	6	2.3	30	8.2	<0.01
Have problems in usual activities	36	36.4	13	4.9	49	13.4	<0.01
Have problems in pain/discomfort	44	44.4	87	32.7	131	35.9	0.04
Have problems in anxiety/depression	48	48.5	201	75.6	249	68.2	<0.01
HIV positive	2	2.0	15	5.6	17	4.7	0.07
	Mean	SD	Mean	SD	Mean	SD	
EuroQOL-5 dimensions (EQ5D) index	0.77	0.23	0.78	0.13	0.78	0.16	0.68
Visual analog scale score (VAS)	82.8	16.7	86.6	12.2	85.6	13.7	0.05

Alcohol consumption is presented in Table 1. About two thirds of clients drank alcohol monthly or more; 36.5 % had three drinks or more on a typical day and 55.5 % ever drank six drinks or more on one occasion. Among 245 drinkers, 62.9 % were hazardous drinkers and 82.0 % were binge drinkers.

Table 2 shows that 90.9 % of rural clients and 96.6 % of urban clients ever had sex (*p* = 0.03). The proportion of respondents having multiple sexual partners in the past 12 months was higher in the urban (48.8 %) than in the rural (13.2 %). About 55.4, 38.3, and 46.1 % respondents did not use condom in the last sex with primary, casual, and commercial sex partners, respectively. Table 2 also describes the proportion of people ever using drug in the urban, which were three times higher than in the rural areas (*p* = 0.02). About 8.7 % urban clients ever injected drug compared to only 2.0 % rural clients *(p* = 0.03). However, there was no difference in current drug use between two groups.

**Table 2:** Sexual risk behaviors and history of drug use among VCT clients in 2013 (*n* = 365)

Factors	Rural		Urban		Total		*p* value
	*N*	%	*N*	%	*N*	%	
Ever had sex	90	90.9	257	96.6	347	95.1	0.03
Number of sex partners (in the last 12 months)							
Not had anyone	34	34.3	18	6.8	52	14.3	<0.01
One sex partners	52	52.5	118	44.4	170	46.6	
2–3 sex partners	6	6.1	94	35.3	100	27.4	
>4 sex partners	7	7.1	36	13.5	43	11.8	
Type of sex partners							
Primary partners	68	68.7	238	89.5	306	83.8	<0.01
Casual sex partners	0	0.0	64	24.1	64	17.5	<0.01
Sex workers	16	16.2	64	24.1	80	21.9	0.10
Non-condom use with last sex							
With primary sex partners (*n* = 305)	42	62.3	127	53.4	169	55.4	0.18
With casual sex partners (*n* = 60)	0	0.0	23	38.3	23	38.3	-
With sex workers (*n* = 76)	16	100.0	19	31.7	35	46.1	<0.01
Ever drug use	4	4.0	34	12.8	38	10.4	0.02
Ever inject drug	2	2.0	23	8.7	25	6.9	0.03
Current drug use	2	50.0	13	38.2	15	39.5	0.65

Table 3 provides information on VCT use amongst respondents. The average number of times of VCT utilization was 1.12 (95 % CI = 0.93–1.31), and there was no difference between rural and urban samples. Spouse and self-motivation were primary reasons for initial VCT used in rural, while peers and media were main motivators in urban (*p* < 0.01). No difference about referring partners to HIV testing services was found between both groups (*p* = 0.26), while the proportion of urban clients being willing to refer other relatives or voluntary peer instructors were significantly higher than rural clients (*p* < 0.05). About 20 and 10.1 % of respondents used outpatient and inpatient care services in the last 12 months, respectively, with no difference between rural and urban.

**Table 3:** Health care and VCT services utilization of respondents in 2013 (*n* = 365)

	Rural			Urban			Total			*p* value
	Mean	95 % CI		Mean	95 % CI		Mean	95 % CI		
VCT service utilization (total times)	1.02	0.68	1.36	1.16	0.92	1.39	1.12	0.93	1.31	0.17
	n	%		n	%		n	%		
Referrer of the first VCT used										
Spouse	22	44.0		14	8.9		36	17.4		<0.01
Peers	5	10.0		48	30.6		53	25.6		
Health workers	2	4.0		21	13.4		23	11.1		
Media	7	14.0		47	29.9		54	26.1		
Self-motivation	9	18.0		22	14.0		31	15		
Parents/relatives	5	10.0		5	3.2		10	4.8		
HIV status (positive)										
Individual	2	4.0		15	9.0		17	4.7		0.12
Spouse/partners	29	29.2		12	4.5		41	11.2		<0.01
Parents	1	1.0		0	0.0		1	0.3		0.1
Brother/sister	5	5.1		3	1.1		8	2.2		0.02
Other relatives	1	1.0		12	5.5		13	3.5		0.2
Refer partners to HIV testing services	54	57.5		166	64.1		220	60.3		0.26
Refer other relatives to HIV testing services	40	43.0		148	57.1		188	51.5		0.02
Volunteer to be a peer Instructor	14	14.9		81	31.2		95	26.8		0.01
Health service use										
Outpatient service use in the last 12 months	15	15.2		58	21.8		73	20.0		0.16
Inpatient service use in the last 12 months	5	5.1		32	12.0		37	10.1		0.05

n=Chi square test

Table 4 presents the reduced multivariate logistic regression models. Clients living with spouse/partners, having problems in anxiety, reporting higher EQ-VAS, using VCT services in urban clinics, and volunteering to be a peer instructor were more likely to have more than two sex partners, while people having HIV-infected spouse were less likely to have multiple partners. Additionally, the interaction between gender and alcohol consumption increased the likelihood of having more than two sex partners. Specifically, clients who reported being male and not hazardous drinkers (adjusted odd ratio (AOR) = 6.21, 95 % CI = 2.62–14.72) or both male and hazardous drinkers (AOR = 4.92; 95 % CI = 2.31–10.47) were more likely to have multiple sex partners compared to group with female only.

**Table 4:** Factors associated with sexual risk behaviors among VCT clients in multivariate regression (*n* = 365; year 2013)

Factors	Having ≥2 sexual partners within 12 months			Not use condom in the last sex with primary partners			Not use condom in the last sex with casual partners			Not use condom in the last sex with sex workers		
	AOR	95 % CI		AOR	95 % CI		AOR	95 % CI		AOR	95 % CI	
Occupation (vs unemployed)												
Self-employed				3.55*	1.33	9.51						
White collars				9.03*	3.24	25.17	2.41*	1.02	5.69			
Workers/farmers				2.66	0.95	7.49						
Other jobs				7.23*	2.05	25.50						
Marital status (vs single)												
Live with spouse/partner	1.90*	1.02	3.52									
Income quintile (vs poorest)												
Poor										2.19	0.89	5.38
Richest	0.48	0.23	1.00									
Have problems in usual activities (yes vs no)										12.94*	1.93	86.96
Have problems in pain/discomfort (yes vs no)							3.42*	1.50	7.78			
Have problems in anxiety/depression (yes vs no)	2.75*	1.44	5.26									
EQ-VAS	1.05*	1.02	1.08	1.02	1.00	1.04						
HIV positive (yes vs no)				0.54*	0.30	0.99						
Interaction between alcohol and gender (vs female + no hazardous drinkers)												
Male + no hazardous drinkers	6.21*	2.62	14.72									
Male + hazardous drinkers	4.92*	10.47								2.35*	1.04	5.33
Number of VCT services uses (times)							1.34*	1.11	1.61			
Not have HIV-positive member in family (yes vs no)				0.42*	0.22	0.78						
Have HIV-infected spouses (yes vs no)	0.10*	0.01	0.86									
Refer relatives to HIV testing services (yes vs no)				1.58	0.88	2.83						
Volunteer to be a peer instructor (yes vs no)	3.73*	1.84	7.55									
Service location area (urban vs rural)	8.04*	3.21	20.14	2.02*	1.05	3.91	6.12*	1.36	27.45			

*AOR*adjusted odd ratio, *EQ-VAS* EuroQOL-visual analog scale

*p < 0.05

Table 4 also shows that employment and urban residence were associated with non-condom use with primary partners, while HIV-positive status and having HIV-positive relatives were associated with increased likelihood of condom use with primary partners. Clients who were white collar reported any problem in pain/discomfort and reported higher number of VCT use, and urban resident were less likely to use condom with casual partners than others. Also, male sex and hazardous drinking were positively associated with non-use condom with commercial partners (AOR = 2.35; 95 % CI = 1.04–5.33).

Factors associated with HRQOL and health care service use are displayed in Table 5. People having pain/discomfort and anxiety/depression problem, drinking three to four drinks, and drinking ≥6 drinks in less than a month were more likely to use inpatient care in the last 12 months. Meanwhile, higher income and having problems in usual activities and pain/discomfort were positive factors for the use of outpatient services.

**Table 5:** Factors associated with HRQOL and health care service use among VCT clients in multivariate regression (*n* = 365; year 2013)

Factors	Inpatient care utilization in the last 12 month			Outpatient care utilization in the last 12 months			EQ-5D Index^a^			EQ-VAS^a^		
	AOR	95 % CI		AOR	95 % CI		Coef	95 % CI		Coef	95 % CI	
Income quintile (vs poorest)												
Poor							0.07	0.01	0.13	4.72	0.38	9.06
Middle				2.53	0.98	6.51						
Rich				4.85*	1.94	12.12						
Richest				3.43*	1.38	8.51						
Have problems in usual activities (yes vs no)				2.43*	1.02	5.78						
Have problems in pain/discomfort (yes vs no)	3.57*	1.46	8.71	5.13*	2.53	10.42						
Have problems in anxiety/depression (yes vs no)	10.91*	1.39	85.66									
HIV status (vs negative)												
Positive										−4.79*	−8.96	−0.63
Frequency of drinking alcohol (vs never)												
≥4 times/week										−9.61*	−18.93	−0.30
Amount of drinks containing alcohol on typical day (vs ≤2 drinks)												
3 or 4	2.85*	1.13	7.23									
5 or 6										5.51	–0.04	11.06
≥10										10.27	−0.08	20.63
Frequency of drinking ≥6 drinks on one occasion (vs never)												
Less than a month	2.11*	1.08	4.15				0.05	−0.01	0.10			
Have ≥2 sex partners in the last 12 months (yes vs no)										6.65*	2.95	10.36
Non-use condom in last sex with primary partners (yes vs no)										4.08	0.41	7.75
Current opiate users (yes vs no)										−11.46*	−19.33	−3.59
Outpatient care utilization in the last 12 months (yes vs no**)**							−0.11*	−0.18	−0.04	−7.69*	−12.00	−3.39
Inpatient care utilization in the last 12 months (yes vs no)							−0.11*	−0.20	−0.02			
Number of VCT services uses (times)										−1.17*	−2.10	−0.25
Not have HIV-positive member in family (yes vs no)										3.98*	0.08	7.87
Refer partners to HIV testing services (yes vs no)							−0.07*	−0.13	−0.02			
Refer relatives to HIV testing services (yes vs no)										4.92*	1.27	8.56

*AOR* adjusted odd ratio, *EQ-5D* EuroQOL-5 dimensions, *EQ-VAS* EuroQOL-visual analog scale

^a^Tobit regression model

*p < 0.05

The results of Table 5 also show that outpatient and inpatient service use as well as referring partners to HIV testing services was associated with lower HRQOL measured using the EQ-5D composite index. In addition, people having HIV-positive status, drinking alcohol ≥4 times/week, current opiate users, utilizing outpatient service, and higher number of VCT use were more likely to report lower EQ-VAS scores. Meanwhile inconsistent condom use with primary partner, not having HIV-positive member in family, and referring relatives to HIV testing were associated with higher EQ-VAS scores among clients.

## Discussion

This study reveals a high prevalence of AUDs among VCT clients and contributes to the growing body of evidence on the association between alcohol abuse and HIV-risk behaviors, lower HRQOL, and increased health care service use [19–23]. In the context of Vietnam where alcohol plays a vital part in the culture, these results provide implications for developing interventions to reduce the burden of alcohol use and HIV/AIDS.

In this study, we observed that 67.1 % VCT clients drank alcohol, of those hazardous drinkers accounted for 62.9 % (42.2 % in the whole sample). This figure was much higher than in general Vietnamese population. For example, a study by Giang et al. (2008) suggested that 25.5 % male and 0.7 % female had AUDs [5]. This prevalence was also much higher than that of patients with HIV/AIDS (30.1 % were hazardous drinkers) [3, 6], male sex workers (29 %), and female sex workers (11 %) [43]. In addition, the association between AUDs and sexual risk behaviors was consistent with previous studies of Vietnam and worldwide [19–23, 44–46]. Having multiple sexual partners [47], inconsistent condom use, and sexually transmitted infections (STIs) were more prevalent among people with AUDs [48, 49]. The prevalence of female having AUDs in this study was much lower than male clients (2.5 vs 61.4 %, respectively). The association between AUD and sexual risk behaviors has been observed in other countries. Carey et al. found that a high level of alcohol consumption may predict the number of sexual partners among female [22]. Another study by Hutton showed that binge drinking was related to risky sexual behaviors and STIs among female, but no association was observed among male [50].

Interestingly, the finding suggested that people who had health problems in usual activities, pain/discomfort and anxiety/depression were more likely to engage in risky sexual behaviors. In the literature, depression and mental health disorders have been found to be associated with unsafe sexual activities [22, 51]. Depression may lead to psychosocial and cognitive impairment [52] that diminishes the ability of patients to prevent risk behavior. Also, risky sexual behaviors may be stigmatized and may lead to depression [51].

In line with previous studies [53, 54], we also found that AUD was associated with decrements in HRQOL and health care service use among VCT clients [55]. The results of this study suggest a need for interventions to reduce the alcohol consumption and facilitate protected sex behaviors as well as drug use abstinence. VCT providers should incorporate the screen of alcohol use problems into their counseling session, offer appropriate information on the harms associated with alcohol use, and provide counseling or treatment service referrals for alcohol as well as other types of substance abuse. This intervention is more important for those who were diagnose with HIV since alcohol and drug abuse are significantly associated with poor adherence and outcomes of HIV/AIDS care and treatment services [6, 7, 11, 12, 31, 32, 56–58]. Second, since VCT clients may experience multiple health problems, including behavioral health issues, health care providers should be aware of complicated health care needs of clients and integrative facilities that provide VCT with general health care, and HIV-related services are important models to improve the efficiency of health services delivery system [28, 59, 60]. Third, findings of this study highlight the multidimensional impacts of high alcohol consumption in Vietnam including not only increased health risks but also higher demand for health care services. Harm reduction policies to reduce alcohol consumption in the country should be contextualized for different settings and focus on raising awareness of community on corresponding health and economic outcomes associated with at-risk alcohol use.

The strength of this study comprises a multiple site sample in two provinces with large HIV epidemics in Vietnam. In addition, the use of an international screening tool for AUD (AUDIT-C), which was validated in Vietnamese settings, has improved the validity of assessment instrument [2, 3]. Also, it helps increase the comparability of the results with other studies in Vietnam and elsewhere. However, there are several limitations that should be acknowledged. First, a convenience sampling limits the representativeness of the study findings. Second, the sample size was small, which may affect the statistical power. Third, information was self-reported, which might lead to recall bias. Finally, the causal relationship between AUD and risk behaviors could not be established due to cross-sectional nature of the study.

## Conclusions

In conclusion, AUD was prevalent, increases the risk of HIV transmission, and diminishes health status of VCT clients. Screening and intervening on alcohol abuse for VCT clients, as well as providing VCT along with general health care services, may improve the efficiency of health service delivery system in Vietnam.
